# How Enrichment Affects Exploration Trade-Offs in Rats: Implications for Welfare and Well-Being

**DOI:** 10.1371/journal.pone.0083578

**Published:** 2013-12-23

**Authors:** Becca Franks, Frances A. Champagne, E. Tory Higgins

**Affiliations:** Department of Psychology, Columbia University, New York, New York, United States of America; University of Houston, United States of America

## Abstract

We propose that a comparative approach to well-being could be the key to understanding ‘the good life.’ Inspired by current theories of human well-being and animal welfare, we designed a novel test of exploration behavior. Environmentally and socially enriched Long-Evans female rats (N = 60) were trained in four simultaneously presented arms of an eight-arm radial-maze. They learned to expect successes in two arms and failures in the other two. After training, 20 animals remained in enriched housing (enrichment-maintenance) while 40 animals were re-housed in standard, isolated conditions (enrichment-removal). Two weeks later, all animals were re-tested in the maze, initially with access to the four familiar arms only. In the final minute, they also had access to the unfamiliar ambiguous-arms. Though both groups showed significant interest in the ambiguous-arms (P<.0001), the enrichment-maintenance group showed a significantly greater exploratory tendency (P<.01) despite having equivalent levels of activity (P>.3). Thus, we show not only that rats will abandon known rewards and incur risk in order to explore, indicating that exploration is valuable in its own right, but also that individuals with (*vs*. without) enriched housing conditions are more likely to engage in such exploratory behavior. This novel test contributes to the body of knowledge examining the importance of exploration in humans and other animals; implications for animal welfare and human well-being are discussed.

## Introduction

Many of the basic patterns of human well-being contain corollaries throughout the animal kingdom from pessimistic cognition in stressed honeybees [1] to longevity in happy orangutans [2]. Human well-being researchers and animal welfare scientists are interested in the same basic questions—what makes life worth living and what environments support such lives—yet more collaboration is possible and likely to be mutually beneficial [3]. In this spirit, we developed a novel test of rat exploration behavior based on theories from animal welfare science and human well-being research.

Similar to other welfare researchers who have made discoveries by applying human psychological constructs to animal behavior [4], [5], we were interested in developing the parallels regarding the role of exploration in well-being and welfare. The motivation to explore is recognized as a key feature in both lines of research 6–10, yet aspects of exploration motivation and its relation to welfare remain unknown. In particular, we wished to 1) investigate a new method for determining the extent to which animals will forgo known rewards and incur possible risks in order to explore their environment and 2) test how manipulations of environmental quality (i.e., housing enrichment) affect this measure of motivational trade-off, and 3) distinguish exploration motivation from approach motivation.

To achieve these aims, we worked with Long-Evans female rats housed in environmentally and socially enriched environments. Rats learned the contingencies in four arms of an eight-arm radial-maze; two of the arms contained successes (food rewards and darkness, i.e. safety for nocturnal animals) and two arms contained failures (mild punishments), while the remaining four arms were blocked from entry. Afterwards, we maintained a subset of the animals in their enriched housing (enrichment-maintenance) and temporarily reduced the housing quality of the remaining animals (enrichment-removal). We chose this manipulation because previous research has shown that temporarily removing enrichment reduces welfare (e.g. starlings [11], mice [12], and rats [13], [14]) without being likely to induce the kinds of extremely negative states that might affect individual differences in approach motivation—i.e., regulatory focus personalities [15]–[17]. We retested all rats in the maze, providing access to the four unfamiliar, ambiguous-arms in the final minute. Thus, we were able to collect data regarding the motivation to explore ambiguous environments, relate that motivation to a manipulation of welfare state, and potentially distinguish it from approach motivation.

## Methods

### Ethics Statement

All procedures were performed in strict accordance with guidelines of the NIH regarding the Guide for the Care and Use of Laboratory Animals and with the approval of the Institutional Animal Care and Use Committee (IACUC) at Columbia University (Protocol Number: AC-AAAC2770). When possible, rats were housed socially and with environmental enrichment to maximize welfare and minimize suffering. When experimental procedures required isolation and the removal of enrichment, animals were checked daily to ensure that they did not show signs of undue stress such as loss of appetite, lethargy, or poor coat condition.

### Animals and Husbandry

Long-Evans female rats (N = 60) were bred and housed with pine-shaving bedding in our animal facility in the Department of Psychology at Columbia University. From the time of weaning (postnatal day 21) until the beginning of the experiments presented here (7 months), rats were group-housed (4/cage) in large cages (38 x 20 x 61 cm) and maintained at a constant temperature and humidity with a 12L:12D light schedule (lights on 9:00 AM). In addition to periodic food enrichment (3-4 times per week of various cereals, fruits, vegetables, nuts, *etc*.), rat chow and water were continuously available. Each cage contained a large opaque plastic insert that provided shelter and environmental complexity.

### Experimental Apparatus

**Figure 1:**
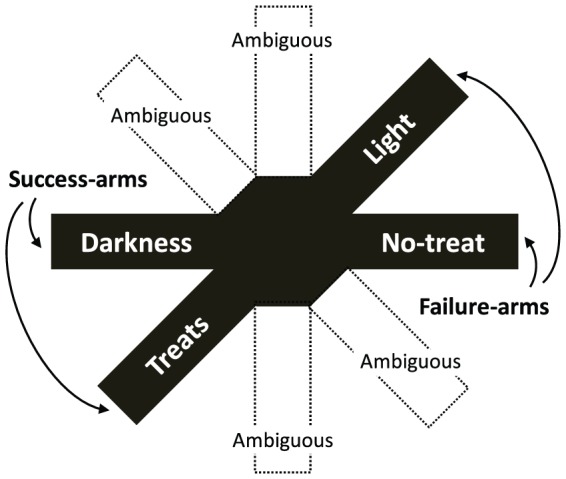
Overhead view of automated-maze. Rats were trained with access to the success- and failure-arms and only had access to the ambiguous-arms in the final minute of the last test.

Rats were tested alone in a radial-arm maze that contained eight arms projecting from a central hub (ScientificDesign; Figure 1). A computer with AnyMaze software recorded the rat’s movement in the maze *via* video camera and automatically activated contingencies when the animal reached the end of an arm. Prior to the experiment reported here, the rats were fully habituated to the test procedures and learned contingencies at the end of four arms while the remaining four arms (marked “ambiguous” in Figure 1) were blocked from entry. Two of the arms were designed to be positively reinforcing *success-arms*: reaching the end of one of the success-arms turned off the overhead light for 30 seconds (dark-arm; prevention/safety success [15]) and reaching the end of the other success-arm released a highly palatable food reward (treat-arm; promotion/gain success [15]). Two of the arms were designed to be negatively reinforcing *failure-arms*: reaching the end of one of the failure-arms turned on the overhead light (light-arm; prevention/safety failure) and reaching the end of the other failure-arm activated a food dispenser mechanism without dispensing a treat (nontreat-arm; promotion/gain failure). In the tests reported here, reaching the end of either of the failure-arms also activated a burst of white noise. The remaining four arms were of ambiguous quality because they were located between a rewarding success-arm and an aversive failure-arm (Figure 1).

### Effect of Removing Enriched Housing

To examine the effect of enrichment on exploration, we tested all animals in the maze before and after an experimental housing manipulation. In the first test, we gathered baseline information regarding overall patterns of treat activations and darkness maintenance as well as individual differences in the motivation to obtain each of these two outcomes—i.e. individual differences in approach motivation: promotion (gain) motivation and prevention (safety) motivation, respectively [15]. In this baseline test, the automated maze provided access to the four arms associated with contingencies (2 success-arms and 2 failure-arms); the ambiguous-arms were not available during baseline tests.

Directly after baseline testing, we randomly assigned two-thirds of the rats (enrichment-removal, N = 40) to be housed in standard laboratory cages with continuous access to food and water but no treats, shelter, gnawing objects, or social companions, all of which are known to be effective enrichment in rats [18], [19]. This environmental manipulation was implemented to induce a relatively poor welfare state in the enrichment-removal group, as has been established by previous research and measured in rats as, for example, reduction in reward sensitivity [11]–[14]. The remaining animals continued under enriched housing (enrichment-maintenance, N = 20). Two weeks after re-housing, all rats were re-tested in the automated maze. During the first four minutes of testing, the maze contingences were as before, but in the fifth and final minute, the ambiguous-arms opened automatically. This minute was the rats’ only exposure to these four arms. The amount of time spent at the end of each ambiguous-arm, the latency to begin exploring, and the number of ambiguous-arms explored was automatically recorded by the AnyMaze software.

### Sucrose Test

To further characterize the relationship between the motivation to explore and (a) welfare and (b) approach motivation, we also tested the enrichment-removal group for sensitivity to the presence of reward or anhedonia [20]. To avoid an experimental confound, we administered the same procedures (two water bottles followed by a 1% sucrose solution the day before testing) to both groups. The animals in the enrichment-maintenance condition were group-housed, however, precluding the collection of individual data points for these animals. As such, we report data on the enrichment-removal group only. During re-housing, all cages contained two water bottles. The day before re-testing in the maze, nearly two weeks later, one of the bottles was filled with 1% sucrose solution. We recorded how much water *vs.* sucrose solution the rat drank over the next 24 hours. The sucrose test measures approach motivation in the sense that sucrose is a rewarding substance that animals tend to approach: greater consumption of sucrose water can therefore indicate greater approach motivation. Simultaneously, as the sucrose solution in this test was diluted to a concentration that has been used previously to detect anhedonia, greater consumption can also indicate better welfare [20]. Thus, our sucrose test provided an opportunity to augment the information we gained regarding the relationship between exploration motivation and approach motivation *and* exploration motivation and welfare.

### Statistical Analyses

To assess individual differences across the two maze tests, we used Pearson’s correlation for normally distributed data (darkness time) and Spearman’s rank correlation for non-normal data (treat counts). We conducted t-tests to investigate the effect of the housing manipulation on behavior in the maze. To generate a composite measure of the motivation to explore (latency to explore, arms explored, change in success-arm time, and time spent exploring), we assessed reliability with Cronbach’s alpha and latent structure with a factor analysis.

## Results

In baseline testing, rats activated an average of 6.51 treats (range: 2, 12), and maintained darkness 32.15% of the time (range 6.25%, 59.48%). Extending previous findings [15], we found these differences in approach motivation—promotion (gains) and prevention (safety), respectively—to be consistent over time despite the housing manipulation. In other words, we found significant correlations between behavior during baseline and the first four minutes of re-testing: treat activations *r_s_* = .59, *p*<.0001 (Spearman’s correlation) and darkness maintenance *r* = .29, *p*<.05 (Pearson’s correlation). Furthermore, as expected, we found no evidence that our housing manipulation affected the mean level of these behaviors (*p*s>.17).

During the first four minutes of the test, when the ambiguous doors were still closed, rats in both conditions spent significantly more of the test time in the success-arms than in the failure-arms: 37.80% and 7.18%, respectively, *t*(59)  = 20.96, *p*<.0001. These times did not vary by condition (*p*s>.20).

**Figure 2:**
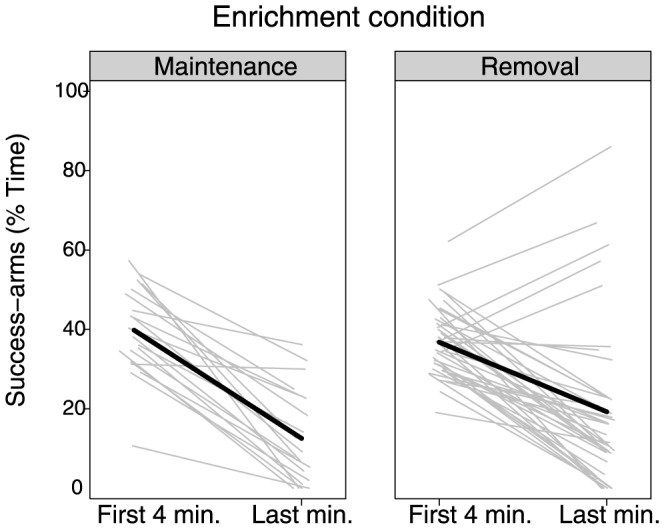
Percentage of time in the success-arms by enrichment condition. Though both groups spent significantly less time in the success-arms after the ambiguous-arms became available, ps<.001, the decrease was significantly greater in the enrichment-maintenance group than the enrichment-removal group, p<.05 (light gray lines: individual responses; thick black lines: average response by condition).

During the fifth and final minute, when the ambiguous doors opened, rats spent an average of 17.02% of the test time in the success-arms, significantly less than during the first four minutes, *t*(59)  = 9.67, *p*<.0001. Importantly, however, the change in behavior was significantly greater in the enrichment-maintenance animals than the enrichment-removal animals: success-arm time dropped by 27.38 percentage points in the enrichment-maintenance group *vs.* 17.53 percentage points in the enrichment-removal group, *t*(58)  = 2.21, *p*<.05 (Figure 2). Moreover, the enrichment-maintenance animals spent significantly more time in the ambiguous-arms than the enrichment-removal animals: 35.67% *vs.* 23.59%, respectively, *t*(58)  = 3.03, *p*<.01. In sum, compared to the enrichment-removal rats, these results suggest that the enrichment-maintenance rats showed significantly greater willingness forgo known rewards and incur possible risks in order to explore the environment.

Reliability and factor analysis indicated that change in success-arm time along with time spent exploring the ends of the ambiguous-arms, latency to begin exploring, and number of arms explored, reliably captured a single latent variable, a = .79, with only a single factor with an Eigen value >1. We therefore used these behaviors to create a composite score as a measure of overall exploratory tendency.

**Figure 3:**
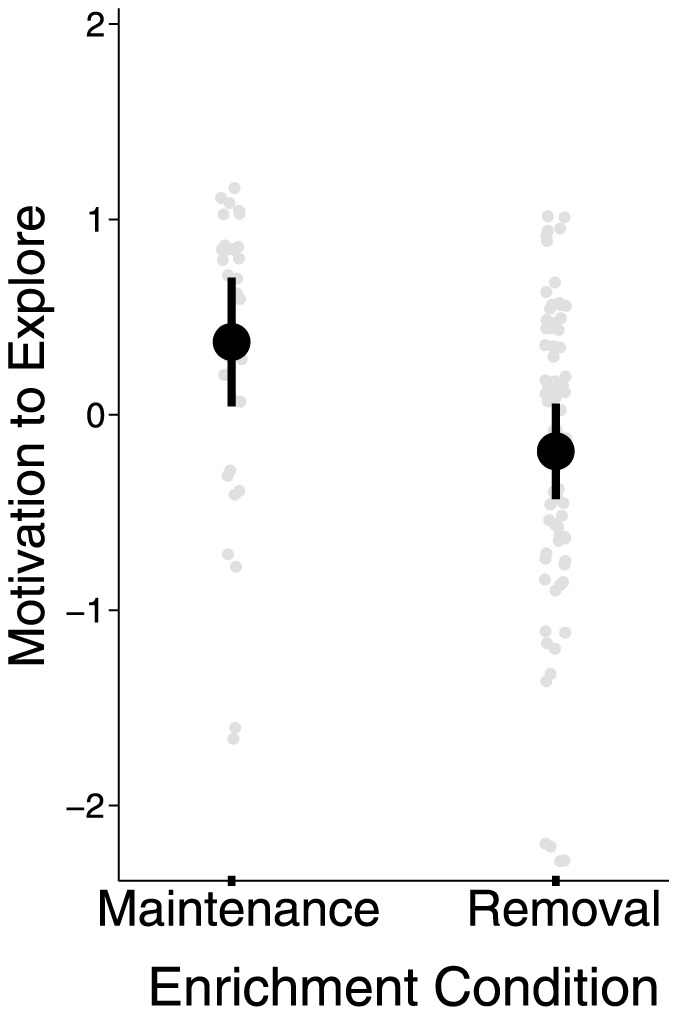
Composite measure of exploration motivation by enrichment condition. Removing enrichment led to a significant reduction in the motivation to explore, p<.01 (light gray dots: individual data points; large black dots: average response by condition; black vertical lines: 95% Confidence Interval).

As predicted, removing enrichment led to a decrease in the motivation to explore, *t*(58)  = 2.74, *p*<.01 (Figure 3). This decrease was not due to a difference in activity: activity level did not vary by condition, *p*>.30, and was unrelated to the composite measure, *p*>.12. Exploratory drive was also unrelated to individual differences in regulatory focus approach motivation [15], [17]: promotion motivation (gains/treats) and prevention motivation (safety/darkness), *p*s>.3. Interestingly, we found evidence suggesting that exploration was *positively* associated with sucrose consumption: within the enrichment-removal condition (our study design did not allow for comparable a metric in the enrichment-maintenance condition—see note in Methods section), the partial correlation between exploratory drive and sugar-water consumption (controlling for water consumption and bottle side) was *r* = .35, *p*<.05.

## Discussion

To test the extent to which animals will forgo known rewards in order to explore environments of ambiguous quality, we measured the behavior of 60 rats in a novel test using an automated maze. Before exploration was possible (when the ambiguous doors were still closed), rats spent an average of 37% of the test time in locations associated with rewarding outcomes. When an opportunity to explore became available (after the ambiguous doors opened), rats spent significantly less time in rewarding locations in favor of spending time investigating the ambiguous environments. These environments were of ambiguous quality because they were located between a rewarding location and an aversive location. Despite the risk of incurring negative outcomes, nearly all the rats (95%) devoted some of the test time to exploration. However, this tendency was not equal in all animals. Two-thirds of the rats (N = 40) underwent a housing manipulation prior to testing; they were removed from their socially and environmentally enriched cages and isolated in standard laboratory cages without enrichment. Compared to the enriched animals, the isolated animals showed a reluctance to forgo known rewards in order to explore ambiguity: they had a smaller decrease in success-arm time and spent less time in the ambiguous-arms.

The patterns of behavior observed in the current study demonstrate several important parallels between human well-being and animal welfare research. First, well-being and welfare research both acknowledge that having desired outcomes is, to some extent, good for welfare [21], [22]. Our housing manipulation leveraged this principle, reducing welfare in part by removing desirable outcomes such as food enrichment. However, both the human well-being and animal welfare literature also recognize that having material resources does not guarantee well-being; a more nuanced view is required. Human research has shown that greater materialism can be correlated with lower well-being [23] and may even lead to reduced well-being [24], while giving away money (the opposite of materialism) can increase happiness [25]. Similarly, animal welfare research has shown that unrestricted access to and desire for food may be harmful to welfare [26], [27]. Thus, both literatures recognize that desirable outcomes, such as money and food, while important, are not the key to the good life [28].

Instead, current models of well-being and welfare emphasize the importance of engagement with challenges and exploration [7], [10], [28]–[30]. Nonhuman animal research has indicated that learning and opportunities to explore enhance welfare [31], [32], which parallels human research indicating that self exploration and knowledge improve well-being [6], [33], [34]. Considering that learning and challenge can improve well-being and welfare, it is somewhat less surprising that despite the risks, humans and other animals tend to seek out or want challenges [6], [10], [28]. Our study furthers this notion by clearly demonstrating that animals will forgo rewards and incur possible risk in order to investigate their environment; the rats in this study show signs of wanting to explore. Thus, beyond desirable outcomes, exploration or opportunities to learn may uniquely contribute to good welfare.

Importantly, however, we also found that the tendency to explore ambiguous locations was less true of animals recently experiencing the removal of environmentally enriching conditions. In humans, research has indicated that people with high (vs. low) well-being are more likely to engage in behaviors that lead to positive emotions, which in turn improve well-being and health [35]. These patterns form a positive-feedback-loop in which well-being can be enhanced and maintained through daily behaviors. The animal welfare literature suggests that a similar pattern could exist in nonhuman animals as well: several lines of research have shown that various species—e.g. rats, parrots, and goats—seek out cognitive/learning challenges [29], [36] and that, in turn, manageable challenges may improve welfare [10]. The research presented here is consistent with these cross-species patterns and may thereby contribute evidence in favor of a positive-feedback mechanism underlying animal welfare. Along with the previous research, our preliminary findings suggest the potential utility of and need for more studies investigating these dynamics.

In addition to addressing possible patterns relating to welfare, we sought to characterize further the motivation to explore. Analyses indicated that the decrease in success-arm time, ambiguous-arm time, latency to begin exploring (reverse coded), and number of ambiguous-arms explored captured a single latent variable, a composite measure of an exploratory motive. Removing enrichment caused a decrease in the motivation to explore that was not attributable to reduced activity levels. Instead, we found that animals housed with continuous enrichment apportion their resources differently than those experiencing a recent removal of environmental and social enrichment; the enrichment-maintenance animals dedicated less of the test time to pursing desirable outcomes (treats and safety) in exchange for more time to explore.

It is possible that the exploration was instrumental, that is, that the ultimate goal of the exploration was to obtain a rewarding outcome or enhance safety. Regardless of whether the behavior was intrinsically or instrumentally motivated, however, it involved engaging in an unfamiliar task and set the conditions to learn something new about the environment. Our data suggest that engaging in novel, exploratory behavior in this test relates to welfare for two reasons: (a) because we found that enriched animals, who were likely to have better welfare, explored more than enrichment-removal animals, who were likely to have worse welfare and (b) because within the enrichment-removal group, we found an inverse correlation between exploration and anhedonia, an indicator of poor welfare [20]. Though our results suggest an exploration-welfare link in this specific test, we are not suggesting that exploration is a diagnostic indicator of good welfare in all cases. For example, compulsive risk taking could involve high levels of (dangerous) exploration arising from behavioral dysregulation or even poor welfare. At a minimum, environmental context and species typical behavior are important moderators of the relationship between welfare and exploratory behavior and other factors could play important roles as well.

It could be argued that animals with poor welfare focused on familiar valuable outcomes (obtaining rewards and avoiding punishments) and not exploration because in their poor condition, they could not afford to divert resources away from certain material benefit. We find little evidence in support of this interpretation, however. First, there was no difference by condition for rewards obtained in the first four minutes of the test (before the ambiguous-arms were available). Second, exploration motivation was *not* inversely related to the motivation for desirable outcomes: there was no significant negative correlation between exploration motivation and individual differences in regulatory focus approach motivations (treat activations and darkness time) [15]–[17]. Indeed, some of the individuals with the highest motivation for these rewarding outcomes also demonstrated the highest motivation to explore. Third, among the enrichment-removal animals, we actually found the opposite pattern: exploratory behavior was *positively* related to sucrose consumption. This final result is intriguing because aside from being a test of how much an animal approaches rewards, the diluted sucrose test has also been used as a measure of anhedonia or poor welfare [20]: animals with poor welfare tend to consume less than animals with good welfare. In combination, therefore, these results support the existence of a distinct exploration motivation that may vary in response to welfare state.

This study contributes to a expanding pool of research suggesting that some of the basic processes of well-being appear conserved across diverse taxa [28]. Many welfare scientists have successfully applied theories from human disciplines such as economics [5] and cognitive psychology [4], but much of the intersection between human well-being and animal welfare remains uncharted. Nevertheless, despite the striking parallels across species, one of the greatest challenges in animal welfare science is to determine how general principles, such as enrichment, take shape in specific species, subspecies, or even individuals. Thus, our enthusiasm for a comparative approach is tempered by the real problem of studying and implementing general principles whose specific instantiations look very different in different species. Despite these challenges, pursuing such lines of research may uncover clues about the fundamental nature and evolutionary significance of “the good life.”
